# Integration of structural variation enhances the resolution of population structure and helps delineate conservation units

**DOI:** 10.1093/aob/mcag062

**Published:** 2026-03-19

**Authors:** Detuan Liu, Rengang Zhang, Yuhang Chang, Yongpeng Ma

**Affiliations:** Yunnan Key Laboratory for Integrative Conservation of Plant Species with Extremely Small Populations, Kunming Institute of Botany, Chinese Academy of Sciences, Kunming 650201, China; Yunnan Key Laboratory for Integrative Conservation of Plant Species with Extremely Small Populations, Kunming Institute of Botany, Chinese Academy of Sciences, Kunming 650201, China; Yunnan Key Laboratory for Integrative Conservation of Plant Species with Extremely Small Populations, Kunming Institute of Botany, Chinese Academy of Sciences, Kunming 650201, China; Yunnan Key Laboratory for Integrative Conservation of Plant Species with Extremely Small Populations, Kunming Institute of Botany, Chinese Academy of Sciences, Kunming 650201, China

**Keywords:** *Rhododendron griersonianum*, resequencing, structural variant, small populations, conservation unit

## Abstract

**Background and Aims:**

*Rhododendron griersonianum* has been classified as Critically Endangered (CR) and listed as a National Key Protected Wild Plant Species in China. Unfortunately, none of its populations were found within nature reserves, leaving them vulnerable to anthropogenic threats. Identifying conservation units is crucial to ensure its survival and recovery.

**Methods:**

We employed whole genome resequencing to identify 5 800 675 single nucleotide polymoprhisms (SNPs) and 11 940 structural variants (SVs). Using these genomic markers, we compared genomic diversity, population clustering and differentiation. By integrating gene flow analysis and demographic reconstruction, we delineated conservation units and formulated management recommendations.

**Key Results:**

Population genomic analysis revealed relatively low genomic diversity in *R. griersonianum*, with values of 1.61 × 10^−3^ for SNPs and 1.36 × 10^−5^ for SVs. Despite the close geographical distances (minimum 5.9 km between populations), we detected limited gene flow (mean *D* = 0.054) and a high inbreeding level (FROH = 0.16), which was estimated to have begun approximately 3500 years ago. The four populations formed three genetic clusters. Population demographic analysis indicated a declining trend in effective population size from 3000 to 1000 years ago. Based on these results, we delineated two management units (MUs), three evolutionarily significant units (ESUs) and three adaptive (putative) units (AUs). We also made recommendations for the future conservation of *R. griersonianum*.

**Conclusions:**

The integration of both SNPs and SVs enhanced the resolution of population structure and refined the delineation of conservation units in this narrowly distributed, small-population species. Our results provide important baseline genetic data, improving our understanding of recent human activities shaping contemporary population structure of small-population species.

## INTRODUCTION

Genetic diversity is central to conservation biology because it enables species to persist and adapt to ambient environments. Extensive evidence demonstrates that species with small, isolated populations are at high risk of extinction (Ralls *et al.*, 2020), and populations with low genetic diversity have been observed to have low survival rates and slow growth rates (Liu *et al.*, 2023). Conversely, greater genetic variability allows wild species to respond to and adapt to changing environments (Hoban *et al.*, 2021), and the conservation of genome-wide genetic variation can improve population viability (Kardos *et al.*, 2021). Over the past 10 years, a growing number of researches have largely moved from using a limited set of microsatellite markers (simple sequence repeats, SSRs) to tapping into thousands of single nucleotide polymorphisms (SNPs) to better inform conservation efforts such as species delineation, detection of hybridization and introgression, identification of conservation units, guidance for conservation translocations, development of conservation breeding programmes and elucidation of the genomic basis of adaptive phenotypes (Liu *et al.*, 2020; Ma *et al.*, 2021, 2022*a*; Wold *et al.*, 2021). However, both SSR and SNP molecular markers quantify genetic variation primarily at the level of a single or a few base pairs, even though resequencing can identify thousands of SNPs.

Structural variants (SVs), which can encompass up to millions of bases, are still poorly characterized in genome evolution (Kou *et al.*, 2020), largely because they were long thought to be rare and computationally difficult to detect (Stuart *et al.*, 2023), and early genetic technologies and reference assemblies lacked sufficient resolution (Yuan *et al.*, 2021). Rapid advances in molecular biology techniques, computational resources and falling sequencing prices have enabled the assessment of SVs in organisms with increasing resolution and accuracy. Growing evidence indicates that SVs represent an important genetic resource, are ubiquitous in organisms, and may have a large influence on a wide range of gene expression and phenotypic traits, which in turn has major impacts on species’ fitness and adaptation (Mérot *et al.*, 2020; Pokrovac and Pezer, 2022; Li *et al.*, 2024). Combined analysis of SNPs and SVs using the same dataset enables us to quantify genetic variation and genetic diversity more accurately and to identify novel adaptive variants (Mérot *et al.*, 2020; Stuart *et al.*, 2023). However, detailed information on the genomic distribution of SVs and their effects on adaptation is lacking (Delmore *et al.*, 2023), especially for non-model endangered plant species.

The genus *Rhododendron* L., which encompasses ∼1200 species worldwide, represents the most diverse genus in Ericaceae. *Rhododendron griersonianum* Balf. f. & Forrest, the only species within subsection *griersoniana* of the genus *Rhododendron*, is one of the most important species for rhododendron hybridization breeding in the international horticultural community. However, owing to its limited population size, *R. griersonianum* has been designated a Plant Species with Extremely Small Populations (PSESP) and classified as a Critically Endangered (CR) species in China. It is also listed in the List of National Key Protected Wild Plant Species under Grade II protection. Recently, the genome of *R. griersonianum* was assembled at the chromosome level, and a population genomic analysis was performed using SNPs from 31 individuals from two populations, 28 from the HQ (Houqiao Town) population and three from the JT (Jietou Town) population (Ma *et al.*, 2021). Our field surveys in 2020 and 2023 revealed that the JT population, comprising just three individuals, had vanished, highlighting the urgent need for conservation efforts for this critically endangered species. Fortunately, our field surveys in 2022 and 2023 led to the discovery of three previously unknown populations in Houqiao Town.

Conservation units (CUs) are critical for determining effective management strategies to maintain the long-term persistence and recovery of populations of an endangered species (Forester *et al.*, 2022). To elucidate the patterns of genome-wide differentiation and gene flow so as to help delineate CUs for the effective recovery of this critically endangered plant species, we resequenced 63 samples from four populations of *R. griersonianum*, and performed population genomic analyses. Genomic variation, including SNPs and SVs, was identified and analysed to compare patterns of distribution, genetic clustering, selection signatures and genomic diversity, which greatly enriched our knowledge of the formation of its small populations and provided fundamental insights into the delimitation of CUs.

## MATERIALS AND METHODS

### Sample acquisition and resequencing

Leaf samples were collected at 5- to 10-m intervals from four populations of *R. griersonianum*, including Dachahe (DCH), Danzha (DZ), Reshuitang (RST) and Guyong (GY) in Houqiao Town, Tengchong City of Yunnan Province, China. As all four populations are found in Houqiao Town, we renamed the HQ population in previous research as the DCH population in this study. A total of 63 samples were collected in this study, 20 from DCH (∼320 individuals), 16 from DZ (∼60 individuals), 15 from RST (∼80 individuals) and 12 from GY (∼70 individuals). Genomic DNA was isolated via a modified CTAB method (Doyle and Doyle, 1987), and then used to construct the short-read library following the manufacturer’s protocol. The libraries were then sequenced individually to an expected depth of 30× on the DNBSEQ T7 platform (BGI Inc., Shenzhen, China).

### Variant detection

Fastp v.0.23.2 (Chen *et al.*, 2018) was chosen to process the raw sequence data to filter out adapter and poor-quality sequences (‘-l 36 --cut_front --cut_tail -c’). High-quality clean reads were then aligned to the *R. griersonianum* genome (Ma *et al.*, 2021) with BWA v.2.1 (Li, 2013) in default settings. Secondary alignments and duplicates were removed with Picard Tools v.2.4.1 (http://broadinstitute.github.io/picard). The bam files were used for downstream analyses.

First, the HaplotypeCaller module of GATK v.4.4.0.0 (Van der Auwera *et al.*, 2013) was used to call variant sites. The raw variants were then processed with the following criteria: (1) INDELs were excluded; (2) genotypes with genotype quality (GQ) less than 20 or genotype depth (DP) less than 5 were considered as missing; (3) sites with more than two alleles were removed; (4) sites with mapping quality (MQ) less than 40 were removed; (5) sites having a maximum missing rate larger than 20 % were removed; and (6) sites with an average sequencing depth larger than 81× (about 2.5× sequencing depth) were marked as missing. After excluding non-variant sites, we obtained 5 800 675 SNPs.

Second, SV calling was performed using three tools, including dysgu (Cleal and Baird, 2022), DELLY (Rausch *et al.*, 2012) and MANTA (Chen *et al.*, 2015), based on the bam file generated above. For dysgu, dysgu run was used to call and genotype SVs across each of the 63 accessions to keep sites with ‘PASS’ labels, and dysgu merge was then used to merge all the 63 vcf files generated. The merged vcf files were used as reference to call variants and keep sites with ‘PASS’ labels (dysgu run --sites merged.vcf). The outputted vcf files were merged. For DELLY and MANTA, SV calling was performed following previous research (Stuart *et al.*, 2023). The SVs called from dysgu, DELLY and MANTA were merged via SURVIVOR v.1.0.7 (Jeffares *et al.*, 2017) with the following parameters: ‘1000 2 1 1 0 30’ (Stuart *et al.*, 2023). That is: the maximum allowed distance of 1 kb was set between predicted break ends, and an SV call could only be retained when it was supported by at least two SV callers; the type and strand of the SVs had to be consistent; and only SV calls with a minimum length of 30 bp were considered. Finally, SVs with allele frequency (AF > 0.3) and SV length ≤1 Mb were retained (Yang *et al.*, 2024). The 1-Mb length threshold was chosen because the vast majority of SVs in this study were shorter than 1 Mb (Supplementary Data Fig. S1).

### Comparison of SNP and SV patterns, genetic diversity and structure

We analysed genome-wide linkage disequilibrium (LD) decay with PopLDdecay v.3.42 (Zhang *et al.*, 2018). To investigate patterns of genomic variation, we calculated the genomic diversity using VCFtools v.0.1.16 (Danecek *et al.*, 2011) setting the parameters to ‘--window-pi 30000 --window-pi-step 10000’ based on the result of LD analysis. We estimated pairwise population differentiation statistics (*F_ST_*) in VCFtools with parameters ‘--fst-window-size 30000 --fst-window-step 10000’. We also used VCFtools to calculate Tajima’s *D* in a 30-kb non-overlapping window. We identified population-private variants in each population. We defined population-private variants as biallelic variants that are fixed (alternate allele frequency, AF = 1) or completely absent (AF = 0) in one population and simultaneously polymorphic (0 < AF < 1) in all others. The analysis described above was conducted using both SNPs and SVs.

To characterize the distribution patterns of SVs and gene regions, we employed SnpEff v.5.1 (Cingolani *et al.*, 2012) to annotate SVs using the *R. griersonianum* genome annotation as a reference (Ma *et al.*, 2021). To quantify the repeat content of the SVs, we performed repeat searches in RepeatMasker v.4.1.7 (Tarailo-Graovac and Chen, 2009). Following previous research, we performed RepeatMasker searches using the first 30 bp of each SV to avoid long SVs returning numerous hits (Stuart *et al.*, 2023). To examine the relationship of global patterns in SNP and SV density, we summarized SNP and SV counts in 1-Mb bins along the genome for all samples with the ‘coverage’ command in BEDTools v.2.28.0 (Quinlan, 2014). We analysed the binned variant densities in R v.4.1.0 (R Core Team, 2013) using the glm() function (family = quasipoisson) to evaluate the relationship between SNPs and SVs.

SNPs were first filtered using VCFtools with the parameters ‘--maf 0.05 --max-missing 0.8’, then pruned using Plink v.1.90b6.21 (Purcell *et al.*, 2007) with the parameters ‘--indep-pairwise 50 10 0.2’ to filter out LD sites, and the generated datasets were referred to as ‘all loci’. We further used two methods to screen for outlier loci, which were considered as putative selected loci. First, genome scans were performed using principal component analysis (PCA) in Pcadapt v.4.3.3 (Luu *et al.*, 2017). Second, we performed a sparse non-negative matrix factorization (sNMF) analysis using LEA v.3.4.0 (Frichot and François, 2015). To decrease the number of false positives, we used the Benjamini–Hochberg method (*P* = 0.05) to correct the significance level when performing pcadapt and sNMF analysis. The union of outlier loci identified by both methods was used as candidate ‘selection loci’. Any locus identified as an outlier was removed from the ‘all loci’ to generate ‘neutral loci’.

SVs were first filtered with the parameters ‘--maf 0.03 --max-missing 0.5’ in VCFtools. Then we performed outlier locus detection for SVs using a method similar to that described above. We conducted GO (Gene Ontology) enrichment analysis on the outlier loci with TBtools (Chen *et al.*, 2020). The significance level for enrichment analysis was set at a *P* value of 0.05.

To investigate genetic structure, we performed structure analysis in Admixture v.1.3.0 (Alexander *et al.*, 2009) with the all, neutral and selection SNP loci. We set the range of genetic cluster (*K*) values to vary from 1 to 4. We also employed PCA to partition the data along their main axes of variation using Plink. We inferred population structure for SVs using the all, neutral and selection SV loci in Admixture and Plink software.

### Testing for gene flow and measuring inbreeding

We employed two tools to detect gene flow events among the four populations of *R. griersonianum*. First, we used TreeMix v.1.13 (Pickrell and Pritchard, 2012) to investigate the split history of lineages and to infer possible migration events. We analysed the addition of 0–10 migration events by generating five replicates for each scenario. We applied a 99.8 % threshold for the proportion of variance explained to determine the optimal number of migration edges that best explained the model (Ma *et al.*, 2022*a*). Second, we used the ‘Dtrios’ command in the Dsuite v.0.5 tool (Malinsky *et al.*, 2021) to compute Patterson’s *D-*statistics (also termed the ABBA-BABA statistic) and *f4*-ratio in the form (((P1, P2), P3), outgroup) to evaluate the presence of introgression between populations based on a phylogenetic tree with *R. facetum* (Ma *et al.*, 2022*b*) as outgroup. The phylogenetic tree was constructed from the vcf file using VCF2Dis v.1.53 (Xu *et al.*, 2025) and ape v.5.8 package (Paradis and Schliep, 2018). Statistical significance of the *D*-statistics was assessed through z scores, with |Z score| > 3 taken as significant evidence of gene flow. Results from ‘Dtrios’ were visualized using the ‘fbranch’ command and the ‘dtools.py’ utility in Dsuite.

To quantify inbreeding levels, we first used the Plink software to detect runs of homozygosity (ROHs) using the following criteria: ‘--homozyg-density 50 --homozyg-gap 1000 --homozyg-kb 100 --homozyg-snp 40 --homozyg-window-het 2 --homozyg-window-snp 40 --homozyg-window-threshold 0.05 --homozyg-window-missing’. We further calculated genome-wide inbreeding coefficients by dividing the total length of ROHs by the genome size for each individual and used one-way ANOVA test to check for significant differences. To quantify the inbreeding history, we also calculated the time (generation) to the common ancestor using the formula: *g* = 100/(2 × *r* × *ROHm*) (Thompson, 2013), where *g* is the number of generations ago, *ROHm* is the mean length of ROHs in megabases (Mb) and *r* is the recombination rate (1 cM Mb^–1^) (Shen *et al.*, 2024).

### Estimation of recent population demography

Demographic history of the four populations was reconstructed to investigate the dynamics of effective population size (*Ne*) in the recent few thousand years using an LD-based method with GONE software (Santiago *et al.*, 2020). The VCF file was transformed into ped and map formats in Plink for each population. GONE analyses were run using default settings except for using 500 independent replicates. A generation time of 10 years was used following previous research (Ma *et al.*, 2021).

### Delineation of conservation units

To guide the management strategies and actions for *R. griersonianum*, we defined an ESU (evolutionarily significant unit) as a cluster of populations using all loci. We use neutral loci to define the MU (management unit) in combination with population structure and geographical barriers. We also define the AU (adaptive unit) using outlier loci (Barbosa *et al.*, 2018; Hohenlohe *et al.*, 2021).

## RESULTS

### Patterns of genetic variants

**Fig. 1.**
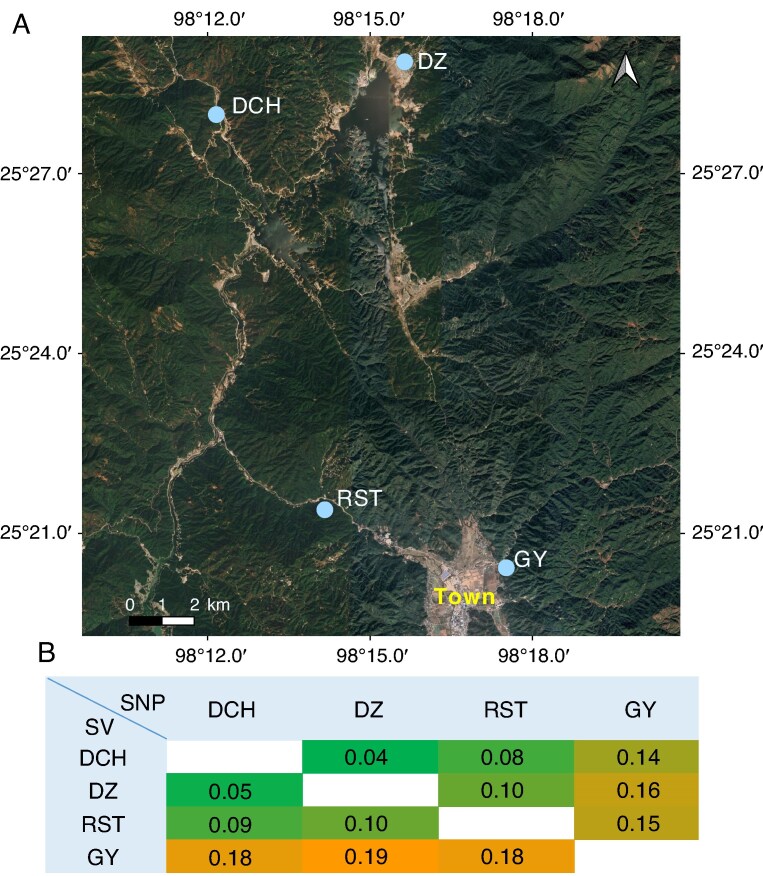
Sample locations and population differentiation matrix. (A) Sample locations of the four populations, created using QGIS v2.0. (B) Population differentiation (*F_ST_*) matrix between populations. Colours and numbers in the cells represent the weighted *F_ST_* values, with green indicating smaller values and orange indicating larger values. Values above the diagonal line were calculated from the SNP dataset, while values below the diagonal line were calculated from the SV dataset. DCH, Dachahe population; DZ, Danzha population; RST, Reshuitang population; GY, Guyong population.

We resequenced 63 individuals sampled from four *R. griersonianum* populations (Table 1; Fig. 1A), generating a total of approximately 11.73 billion raw reads (∼41×, Supplementary Data Table S1). After quality control, ∼1752 Gb of clean data were obtained, 96 % of which were successfully aligned to the reference genome. A total of 5 800 675 SNPs were obtained in the 63 individuals after hard filtering. We also identified a total of 39 290 SVs from the 63 individuals. After filtering out SVs longer than 1 Mb, the final SV dataset contained 11 940 SVs, including 9142 deletions (DEL), 384 duplications (DUP), 1377 insertions (INS), 156 inversions (INV) and 881 translocations (TRA).

**Table 1.** Sample information, genome-wide diversity (*π*) and Tajima’s *D*-statistics estimated with SNPs and SVs.

Population	Sample number	SNP			SV		
		Private number	*π*	Tajima’s *D*	Private number	*π*	Tajima’s *D*
DCH	20	88 842	1.53E-03	0.21	136	1.36E-05	1.17
DZ	16	141 016	1.53E-03	0.25	341	1.36E-05	1.08
RST	15	328 706	1.47E-03	0.42	685	1.35E-05	1.06
GY	12	759 533	1.27E-03	0.51	729	1.39E-05	0.88
Total	63		1.61E-03	0.15		1.36E-05	1.48

DCH, Dachahe population; DZ, Danzha population; RST, Reshuitang population; GY, Guyong population.

The LD decay plot exhibited a rapid decline in LD correlation (*r*^2^), which dropped to half its maximum at around 30 kb (Supplementary Data Fig. S2). The overall genomic diversity (*π*) was relatively low for both SNPs and SVs, with a significantly higher diversity for SNPs compared to SVs (Table 1). For the SNP genetic variants, the genomic diversity was lower in the GY population than in the other populations (Table 1). Conversely, the GY population had a slightly higher genetic diversity than the other three populations when assessed on SVs (Table 1). The DCH population had a much lower number of private SNPs (88 842), and the GY population had the greatest number of private SNPs (759 533) (Table 1). When comparing the number of private SVs, we observed a consistent pattern across the four populations (Table 1).

We also estimated Tajima’s *D* and genetic differentiation based on SNPs and SVs. Tajima’s *D* values were positive (Table 1). The overall genetic differentiation, as quantified by *F_ST_* estimates, was low when calculated using both SNPs and SVs; however, it was higher when calculated using SVs than SNPs (Fig. 1B). Among the four populations, the GY population had the highest *F_ST_* distance compared to the others, indicating a higher degree of genetic differentiation.

**Fig. 2.**
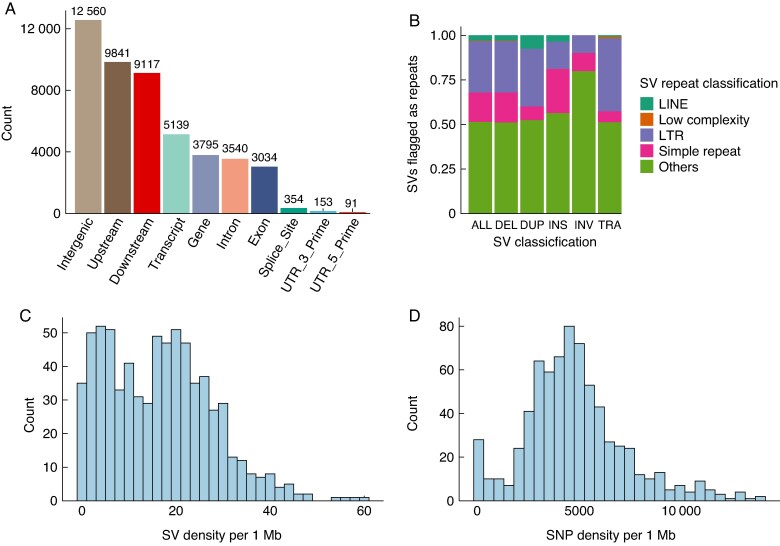
Patterns of genetic variation in *R. griersonianum*. (A) Number of SVs overlapping with different genomic regions relative to gene models, including intergenic, CDS, intron, upstream (<1 kb) and downstream (<1 kb) regions for SVs. (B) Proportion of repeat types across SVs. (C) Histogram of SV density in 1-Mb windows. (D) Histogram of SNP density in 1-Mb windows.

Among the SV classes, DEL was the predominant type, comprising approximately 74 % of the total SVs (Supplementary Data Table S2). The result of SV annotation revealed that 12 560 (26.35 %) intergenic regions of genes overlapped with SVs, 3795 (7.96 %) genes overlapped with SVs, and 3034 (6.73 %) and 3540 (7.43 %) genes contained SVs in their exons and introns, respectively (Fig. 2A; Table S2). We also annotated the repeat elements that overlapped with the SVs and found that the majority consisted of the other repeats, including DNA, rRNA, small nuclear RNA (snRNA), etc. (Fig. 2B). Long terminal repeats (LTRs) were the second most common category (Fig. 2B). Overall, there was a positive correlation between the density of SNPs and SVs, as indicated by a linear model with a *P* value of less than 0.001 (*t* = 19), and a correlation coefficient of 0.61 (Fig. S3). Comparative analysis of the density patterns between SVs and SNPs revealed that SVs were significantly less frequent, typically occurring at 0–30 per Mb, whereas SNPs were denser at 3000–6000 per Mb along the chromosomes (Fig. 2C, D).

### Selection signature and population structure

A total of 738 outlier SNPs and 260 outlier SVs were identified. These outlier loci were considered as putatively selected. Functional annotation and GO enrichment analysis showed that many genes carrying outlier SNPs were significantly enriched with genes associated with lipid metabolism process (GO:0006629, GO:0044255, Supplementary Data Table S3), while many genes carrying outlier SVs were significantly enriched with genes involved in response to abiotic stimulus (GO:0009628) and response to water deprivation (GO:0009414, Table S4).

**Fig. 3.**
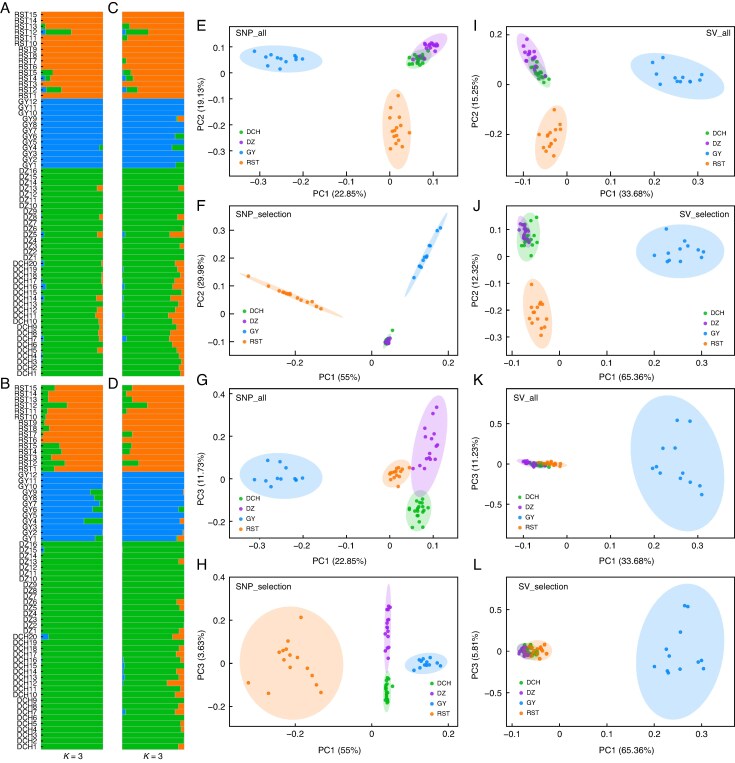
Population structure analyses of *Rhododendron griersonianum.* (A) Admixture plots of all SNPs at *K* = 3. (B) Admixture plots based on selected SNPs at *K* = 3. (C) Admixture plots based on all SVs at *K* = 3. (D) Admixture plots based on selected SVs at *K* = 3. (E, G) Principal component analysis (PCA) plots of PC1 vs PC2 and PC1 vs PC3 based on all SNPs. (F, H) PCA plots of PC1 vs PC2 and PC1 vs PC3 based on selected SNPs. (I, K) PCA plots of PC1 vs PC2 and PC1 vs PC3 based on all SVs. (J, L) PCA plots of PC1 vs PC2 and PC1 vs PC3 based on selected SVs. DCH, Dachahe population; DZ, Danzha population; RST, Reshuitang population; GY, Guyong population.

The result of Admixture analysis indicated that the optimal *K* = 3 using all SNP loci (Supplementary Data Fig. S4A), with the DCH and DZ populations clustering together, and the RST and GY populations each forming one cluster (Fig. 3A). Similar patterns of genetic clusters were detected when using outlier SNPs (Fig. 3B; Fig. S4B). However, the SV-based Admixture analysis showed similar but more mixed signals (Fig. 3C, D; Fig. S4C, D).

Additional PCA further supported the separate genetic clustering of the RST, GY, DCH and DZ populations into three separate clusters using both all SNP loci (Fig. 3E, G) and all SV loci (Fig. 3I, K). Specifically, the PCA clustering analysis showed that the DCH population could be separated from the others by PC3 when using all SNPs (Fig. 3G), but not when based on selected SVs (Fig. 3K). The total variation explained by the first three principal components (PC1, PC2 and PC3) was 22.85 %, 19.13 % and 11.73 % for all SNPs, and 33.68 %, 15.25 % and 11.23 % for all SVs, respectively. This pattern was consistent when neutral SNP loci and neutral SV loci were used (Supplementary Data Fig. S5). When focusing on selected loci, the population structure also showed inconsistent patterns between SNPs (Fig. 3F, H) and SVs (Fig. 3J, L). Specifically, the PCA clustering analysis showed that both the RST and GY populations could be separated from the others by PC1 when using selected SNPs (Fig. 3F, H), while only the GY population could be separated from the others by PC1 (Fig. 3J, L), and the RST population could only be separated from the others by PC2 (Fig. 3J) when based on selected SVs. The variation explained by PC1, PC2 and PC3 was 55 %, 29.98 % and 3.63 % for selected SNPs, and 65.36 %, 12.32 % and 5.81 % for selected SVs, respectively.

### Gene flow and inbreeding level

**Fig. 4.**
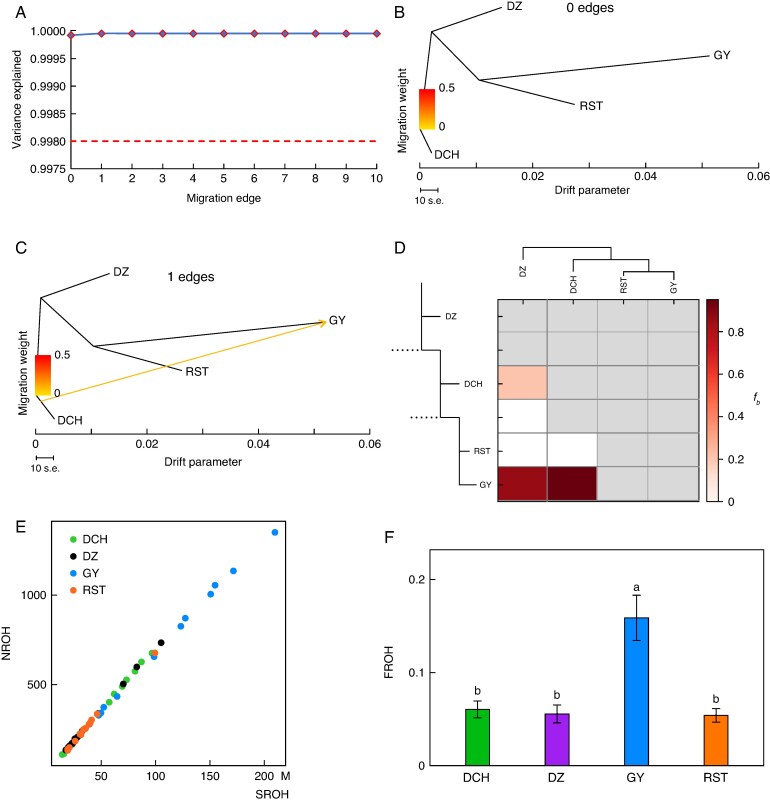
Levels of gene flow and inbreeding among the four populations of *R. griersonianum.* (A) Proportion of variation explained by each migration edge. The red dotted line indicates the 99.8 % variation threshold. (B) Gene flow inferred by TreeMix with 0 migration edges. (C) Gene flow inferred by TreeMix with 1 migration edges. Arrows indicate the direction of gene flow, coloured according to the percentage (weight) of alleles from the source population. (D) Heatmap for pairwise *f-branch* (*f*_b_) statistics calculated using Dsuite software among the four populations. Coloured squares indicate a significant excess of SNPs shared between branches due to gene flow. Cells in grey indicate comparisons that cannot be made. Dotted lines indicate ancestral lineages. The colour scale at right indicates values of alleles shared between the tree branch on the vertical axis and the population on the horizontal axis. (E) Sum of the total length of ROH (SROH) vs the total number of ROH (NROH). (F) Fractions of runs of homozygosity (ROH) greater than 100 kb for each population. Different letters indicate significant differences between groups. DCH, Dachahe population; DZ, Danzha population; RST, Reshuitang population; GY, Guyong population.

The results of TreeMix analysis showed that the model with zero migration edges could explain 99.99 % of the covariance in allele frequencies (Fig. 4A, B). We also fitted the model with one migration edge, which detected gene flow from the DCH population to the GY population (Fig. 4C). Consistent patterns were obtained from the other models fitted with 2–10 migration edges. The *D*-statistics and *f*4-ratio analysis revealed signals of gene flow between the DCH and GY populations (*D* statistic = 0.070, *Z* score = 9.93, *f*4-ratio = 0.75), between the GY and DZ populations (*D* statistic = 0.073, *Z* score = 10.32, *f*4-ratio = 0.86), and between the DCH and DZ populations (*D* statistic = 0.037, *Z* score = 6.72, *f*4-ratio = 0.43) (Fig. 4D; Table 2).

**Table 2.** Result of ABBA-BABA tests in Dsuite.

P1	P2	P3	*D* statistics	Z-score	*P*-value	*f*4-ratio	BBAA	ABBA	BABA
DCH	GY	DZ	0.036	5.68	1.35E-08	0.75	132 414	124 048	115 388
RST	DCH	DZ	0.037	6.72	1.79E-11	0.43	121 792	121 635	112 945
RST	GY	DCH	0.070	9.93	2.30E-16	0.95	132 177	128 840	111 971
RST	GY	DZ	0.073	10.32	2.30E-16	0.86	139 910	128 208	110 856

DCH, Dachahe population; DZ, Danzha population; RST, Reshuitang population; GY, Guyong population.

To investigate the degree of inbreeding, we analysed the distribution of ROH length and number among these four populations. A total of 22 869 ROHs were detected, with the GY population having a higher number than the others (Fig. 4E). Most ROHs were shorter than 1 Mb, with only two exceptions longer than 1 Mb in the GY population. On average, the ROHs were ∼143 kb in length (Supplementary Data Table S5), with shorter ROHs (<200 kb) being predominant and accounting for ∼90 % (20 489/22 869) of the total number in all four populations. Shorter ROHs are linked to ancient inbreeding (Ceballos *et al.*, 2018). The estimated time of inbreeding using ROH length was approximately 350 generations ago, which was approximately 3500 years ago, assuming 10 years per generation (Ma *et al.*, 2021). In addition, we evaluated the inbreeding coefﬁcient by assessing the proportion of ROHs longer than 100 kb (FROH). We found that the level of inbreeding in the GY population (FROH = 0.16) was significantly higher than in the other populations (Fig. 4F).

### Recent demography

**Fig. 5.**
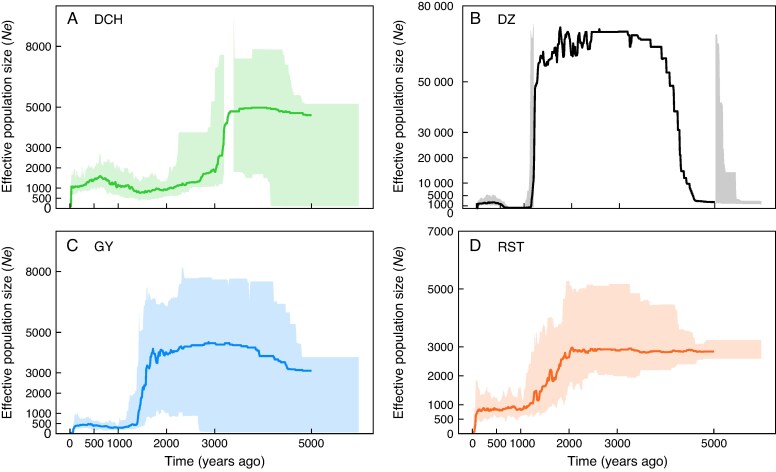
Estimation of effective population size over the last 500 generations using GONE software. (A) Changes in effective population size (*Ne*) of the DCH population. (B) Changes in effective population size (*Ne*) of the DZ population. (C) Changes in effective population size (*Ne*) of the GY population. (D) Changes in effective population size (*Ne*) of the RST population. The dark colour line indicates the mean estimate of effective population size (*Ne*), and the light background colour corresponds to the upper and lower limits of the 95 % confidence intervals. DCH, Dachahe population; DZ, Danzha population; RST, Reshuitang population; GY, Guyong population.

The results of GONE analysis showed an overall declining pattern in *Ne* across the four populations over the last 500 generations (Fig. 5). Specifically, the DCH and RST populations showed a gradual and more continuous decline in *Ne* starting ∼3000 years ago (Fig. 5A) and ∼2000 years ago (Fig. 5D), respectively. In contrast, the DZ and GY populations showed a significant reduction in *Ne* starting ∼1000 years ago (Fig. 5B) and ∼2000 years ago, respectively.

### Conservation units

**Fig. 6.**
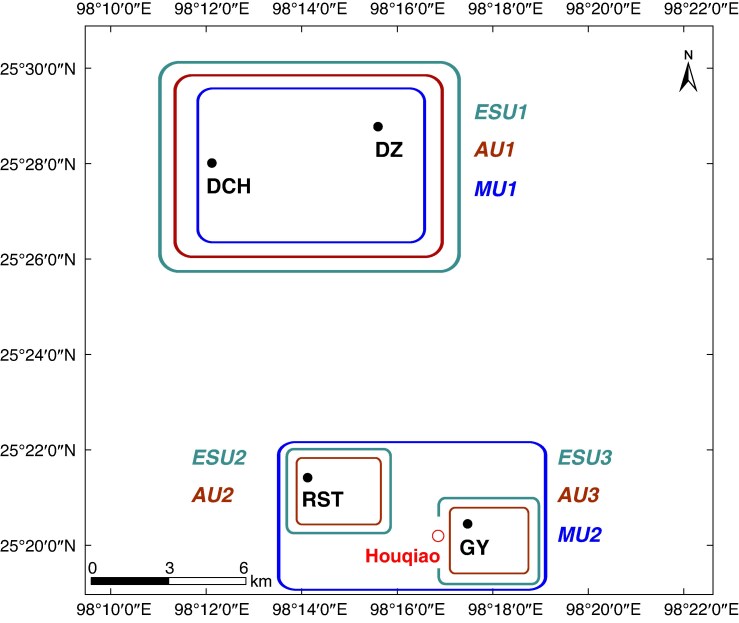
Designation of conservation units for *R. griersonianum*. Genome-wide variation estimated using all loci grouped populations into evolutionarily significant units (ESUs). Neutral and selected variation facilitated the delineation of management units (MUs) and adaptive units (AUs), respectively. The red circle represents the location of Houqiao Town. DCH, Dachahe population; DZ, Danzha population; RST, Reshuitang population; GY, Guyong population.

Population cluster analysis using all loci in Admixture and PCA identified three genetic clusters, which we then categorized into three ESUs: ESU1 included the DCH and DZ populations, ESU2 included the RST population, and ESU3 included the GY population (Fig. 6). Similarly, based on the population structure results using selection loci, we grouped the DCH and DZ populations into one adaptive unit (AU1), the RST population into AU2 and the GY population into AU3 (Fig. 6).

Population structure analysis using both SNPs and SVs at neutral loci revealed that the DCH and DZ populations cluster together. The DCH and DZ populations were administratively managed under the Danzha Management Station, while the RST and GY populations belonged to another station and were in close proximity. Based on these findings, we delineated the DCH and DZ populations as one MU and the RST and GY populations as a separate MU (Fig. 6).

## DISCUSSION

### Contrasting patterns of SNP and SV loci

SVs are key components of intraspecific genetic variants, occurring in a variety of forms and serve as pervasive drivers of ecological adaptation and evolutionary responses (Mérot *et al.*, 2020). Due to the extremely small populations and restricted distribution range, *R. griersonianum* is in urgent need of rescue. To characterize genetic variants and elucidate the characteristics of population structure, thereby informing specific conservation measures, we performed whole-genome resequencing of 63 individuals of *R. griersonianum* from four populations and performed integrative population genomic analyses based on SNPs and SVs called from short read sequencing data in the current research. We found that the overall genome-wide diversity estimated from SNPs was higher than that from SVs, and the patterns of genetic differentiation and population structure revealed by these two types of genetic variants were not entirely consistent, suggesting that SVs may capture additional population structure and genetic diversity compared to SNP-only approaches (Stuart *et al.*, 2023). Importantly, we observed that the GO enrichment results of selective SVs and selective SNPs were largely distinct, suggesting that the two types of genetic variants may be associated with different functional categories and potentially subject to different selective pressures. The differences between SNP- and SV-based population structures may reflect distinct evolutionary dynamics, possibly including differential selective pressures acting on these variants. Consequently, conservation strategies based solely on SNP data might overlook critical genetic partitions. To fully assess population structure, it is necessary to examine SVs among populations within a taxon and to further investigate the structure among frequently studied SNPs (Stuart *et al.*, 2023).

The results of GO enrichment analysis on the outlier SNP loci and SV loci further suggest that different types of genetic variants are likely to have different functions within a population. This finding underscores the complexity of evolutionary processes and highlights that different genetic elements may be influenced by different selective pressures, which in turn are likely to result in different genetic landscapes, thereby generating heterogeneous adaptive potential. Previous work suggested that SVs are typically found at lower frequencies than SNPs in the domestication of Asian rice (*Oryza sativa*), probably due to their potentially harmful effects on the organism (Kou *et al.*, 2020). Conversely, SVs may play an important role in shaping ecological phenotypes and facilitating local adaptation in *Arabidopsis thaliana* (Kang *et al.*, 2023). Critically, for rare and small population species like *R. griersonianum*, where nucleotide diversity is severely eroded, SVs may represent a vital reservoir of functional variation that could support resilience and local adaptation. Exclusively SNP-based conservation frameworks may overlook ecologically relevant adaptive variation of SV loci.

Integrating SVs into conservation genomics could enable more accurate delineation of CUs, particularly when SNP-based population clustering signals are weak, especially for narrowly distributed endangered species harbouring unique functional SV variants. We therefore advocate for an integrative dual-marker conservation genomics framework incorporating both SNPs and SVs for evidence-based conservation planning of endangered species, as it captures more genomic architecture heterogeneity, adaptive genetic variation, and landscape-level population connectivity. Future research efforts should prioritize functional validation of candidate SVs through examining differential gene expression patterns to further elucidate their roles in the adaptation of *R. griersonianum*.

### Low genetic differentiation and limited gene flow

The four populations of *R. griersonianum* showed limited genetic differentiation. This pattern could be interpreted in two possible scenarios. First, the limited genetic differentiation could be due to recent human-induced fragmentation and isolation. Previous research has shown that *R. griersonianum* recovered from a bottleneck event approximately 15000 years ago and subsequently maintained a large *Ne* to the present day, and its small census sizes are likely caused by human activities (Ma *et al.*, 2021). The positive Tajima’s *D* values are likely a consequence of population bottlenecks. Our GONE analysis also suggests a dramatic decline in *Ne* over the last 3000–1000 years. We therefore inferred that *R. griersonianum* has recently declined and isolated into several small populations. In large populations, genetic drift is generally too slow to produce noticeable genetic differences, even over many generations (Keller *et al.*, 2004). Genetic similarities may be due to the common ancestry of recently isolated populations (Zhao *et al.*, 2023). Second, despite their separation into four populations, the four populations of *R. griersonianum* are geographically close and inhabit ecologically similar environments and are likely to be subject to analogous selection. Furthermore, recurrent bottlenecks can lead to extreme and rapid genetic drift, which may mask the signatures of selection.

Despite their geographical proximity, we detected very weak gene flow among the four populations with Treemix, ABBA-BABA tests and *f*4-ratio statistics. Structure analysis also revealed some signals of genetic mixture, with the DCH and DZ populations clustered together, and they shared some admixture with the RST and GY populations. There are two main interpretations of this observation. First, previous research has shown that *R. griersonianum* has a pollinator-dependent mixed mating system, with butterflies such as *Papilio helenus* and *P. bianor* being effective pollinators, while it suffers from pollination limitation due to pollinator scarcity and exhibits inbreeding avoidance pollination syndromes (Liu *et al.*, 2025). It has been documented that some species of the genus *Papilio* have flight ranges of less than 2 km (Pryke and Samways, 2001). The paucity of pollinators and their limited flight distances may limit gene flow between the four populations. In addition, butterflies are sensitive to human activities and habitat fragmentation, which may affect their flight behaviour, abundance, distribution patterns and flight range (Yu *et al.*, 2023), further reducing or limiting mate availability and gene flow between *R. griersonianum* populations. Second, the four populations of *R. griersonianum* are separated by villages, roads, buildings, a reservoir and farmland, which act as significant geographical barriers that may reduce the potential for genetic exchange and impede gene flow.

### Conservation implications

With low genomic diversity, limited gene flow, small population sizes and continued pressure from human activities, *R. griersonianum* has a heightened extinction risk. Based on our findings, we propose the following conservation measures to prevent the extinction of this small-population species.

(1) Understanding the genetic factors that cause the decline of endangered populations is crucial for effective biodiversity conservation. Genetic variation and inbreeding depression are two major issues in conservation biology (Kardos *et al.*, 2023). Small, isolated populations are at heightened risk of demographic collapse due to low genomic diversity, genetic drift and high levels of inbreeding. Consistent with the findings of Ma *et al.* (2021), we found that the three newly discovered populations had lower genomic diversity. Limited genomic diversity could weaken their adaptability to environmental change. Facilitating gene flow among isolated small populations constitutes an effective strategy to increase fitness by raising genomic diversity and minimizing inbreeding, a process commonly termed genetic rescue (Wang *et al.*, 2022). Ma *et al.* (2021) emphasized the importance of identifying new populations for genetic rescue. Fortunately, the discovery of three additional populations in Houqiao Town provides a significant opportunity for this. However, limited gene flow was detected between the four populations of *R. griersonianum*, despite the relatively close geographical distance, with the shortest distance being 5.9 km between the DCH and DZ populations, and the longest being 16.6 km between the DCH and GY populations. Therefore, artificial supplemental pollination would be required to facilitate gene exchange, promote outcrossing and increase genetic diversity, which can further improve fruit set and seed production rates. Notably, the GY population exhibited the highest genetic differentiation among all the populations, and holds the potential to mask more deleterious variants. It also harbours the largest number of private genetic variants. However, this population faces the highest extinction risk due to its close proximity (<150 m) to Houqiao Town, which may expose it to increased anthropogenic disturbance. We therefore recommend prioritizing the use of pollen from the GY population for artificial pollination in the other three populations to enhance genetic diversity and facilitate genetic rescue.

(2) Accurate identification of CUs is critical to the effectiveness of conservation efforts, as it provides information on species status and guides the development of management strategies crucial for the recovery and long-term survival of endangered populations (Forester *et al.*, 2022; Lehnert *et al.*, 2023). Rapid advances in genomics have revolutionized the delineation of CUs for rare or endangered species by enabling the identification of adaptive genetic variation (Funk *et al.*, 2012). In this study, we delineate CUs using all loci, neutral loci and outlier loci of SNPs and SVs. Our population structure analysis revealed distinct genetic clustering of the RST, GY, DCH and DZ populations as three separate clusters using different datasets. These findings highlight that significant population structure can exist within taxa with a limited narrow distribution, highlighting the need to identify fine-scale genetic structure to refine the delineation of CUs in narrowly distributed plants, and thus enhancing conservation approaches for their evolutionary potential of threated plants (Li *et al.*, 2023). In particular, the GY population showed the greatest genetic divergence from the other three populations, indicating its unique genetic components. Our consistent results from analyses incorporating SNPs and SVs at different loci strongly suggest that the GY population should be recognized as a separate ESU, AU and MU, due to its genetic independence. Given the disappearance of the JT population, it is imperative that *in situ* conservation plots or protection sites be established as a high priority to protect the remaining wild populations and their native habitats. In this sense, cross-pollination between the GY and other populations is recommended to increase genetic diversity and support the resilience of this endangered species.

(3) The GY population grows along roadsides and near residential buildings in Houqiao Town, with a direct distance of less than 150 m. This population is at high risk of extinction and requires increased conservation awareness and proactive conservation strategies. Our genetic analysis revealed that the GY population harbours the largest number of private SNPs and SVs, highlighting its unique genetic composition and significance. To prevent the imminent extinction of this genetically valuable population, we strongly recommend prioritizing seed collection from this population, which will not only safeguard its unique genetic diversity but also provide material for *ex situ* conservation efforts and future reintroduction programmes.

(4) To mitigate the adverse effects of human activities such as reservoir construction, transportation and livestock breeding on the natural habitats of *R. griersonianum*, which result in the destruction of seedlings and hinder the regeneration of the population, we recommend to germinate seeds and propagate seedlings in a controlled nursery environment. Once the seedlings have reached a suitable stage of development, reintroduce them into natural habitats, thereby increasing their chances of survival and helping to maintain the population of the species.

## CONCLUSIONS

In summary, our whole-genome resequencing analysis of 63 individuals from four populations of *R. griersonianum* revealed relatively low genetic diversity, weak genetic differentiation and limited gene flow among the four populations. We found inconsistent patterns of population structure and selection signatures when using SNPs and SVs, and SVs are informative markers capable of capturing additional population structure and genetic variants compared to SNP-only approaches, highlighting the importance of incorporating SNPs and SVs when addressing conservation efforts for endangered plant species with extremely small populations and narrow ranges. Demographic analysis suggests a declining trend in the effective population size over the past 300–100 generations. Based on these findings, we delineate two MUs, three ESUs and three (putative) AUs. Collectively, these findings could provide valuable insights for the future conservation and management of *R. griersonianum*.

## Supplementary Material

mcag062_Supplementary_Data

## Data Availability

Raw sequence data have been deposited in the China National Center for Bioinformation (CNCB, https://ngdc.cncb.ac.cn) under the BioProject accession number PRJCA034380.
