# Supplementary material for: Integration of structural variation enhances the resolution of population structure and helps delineate conservation units
Source: Ann Bot. 2026 Mar 19;137(7):2095–107. doi: 10.1093/aob/mcag062 (PMC13319329; doi:10.1093/aob/mcag062)
Supplement: mcag062_Supplementary_Data [file mcag062_supplementary_data.zip › Supplementary data 1.docx]

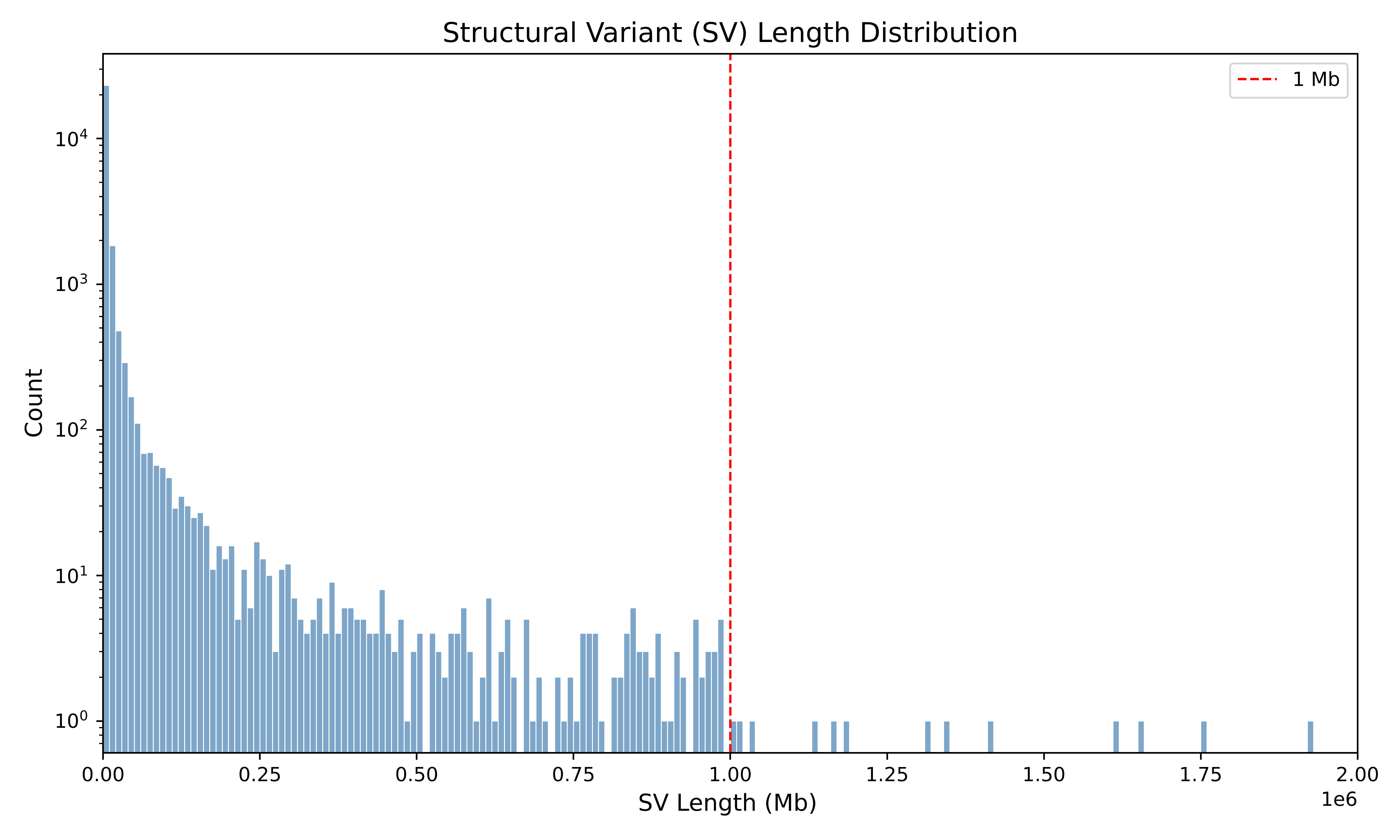


**Fig. S1** Distribution of structural variant (SV) lengths. A subset of SVs—such as translocations (TRA)—have no change in DNA length (SVLEN = 0), resulting in zero length in the distribution.


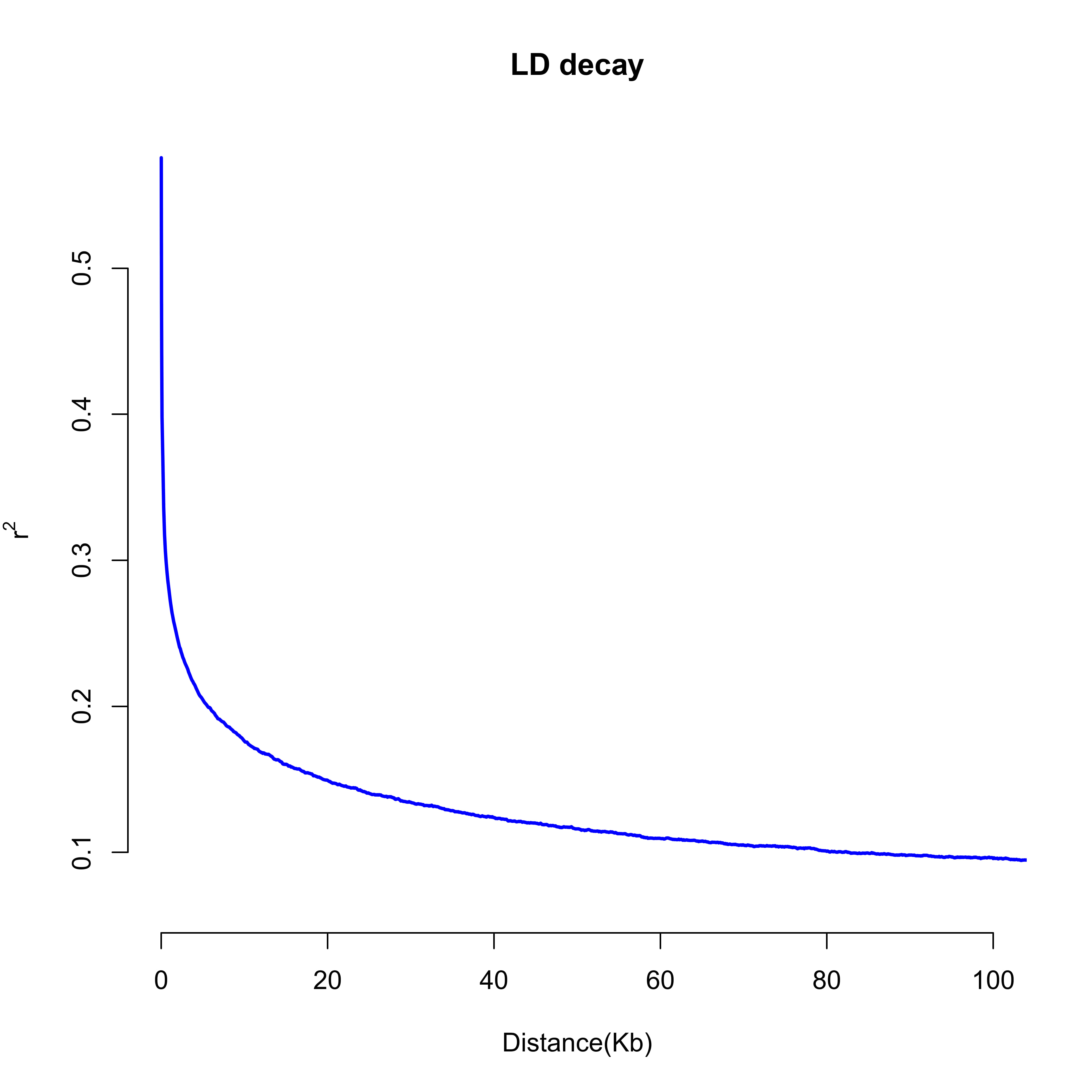


**Fig. S2** Plot of LD decay. The horizontal axis indicates the distance between two points on the genome, and the vertical axis indicates the linkage disequilibrium coefficient.


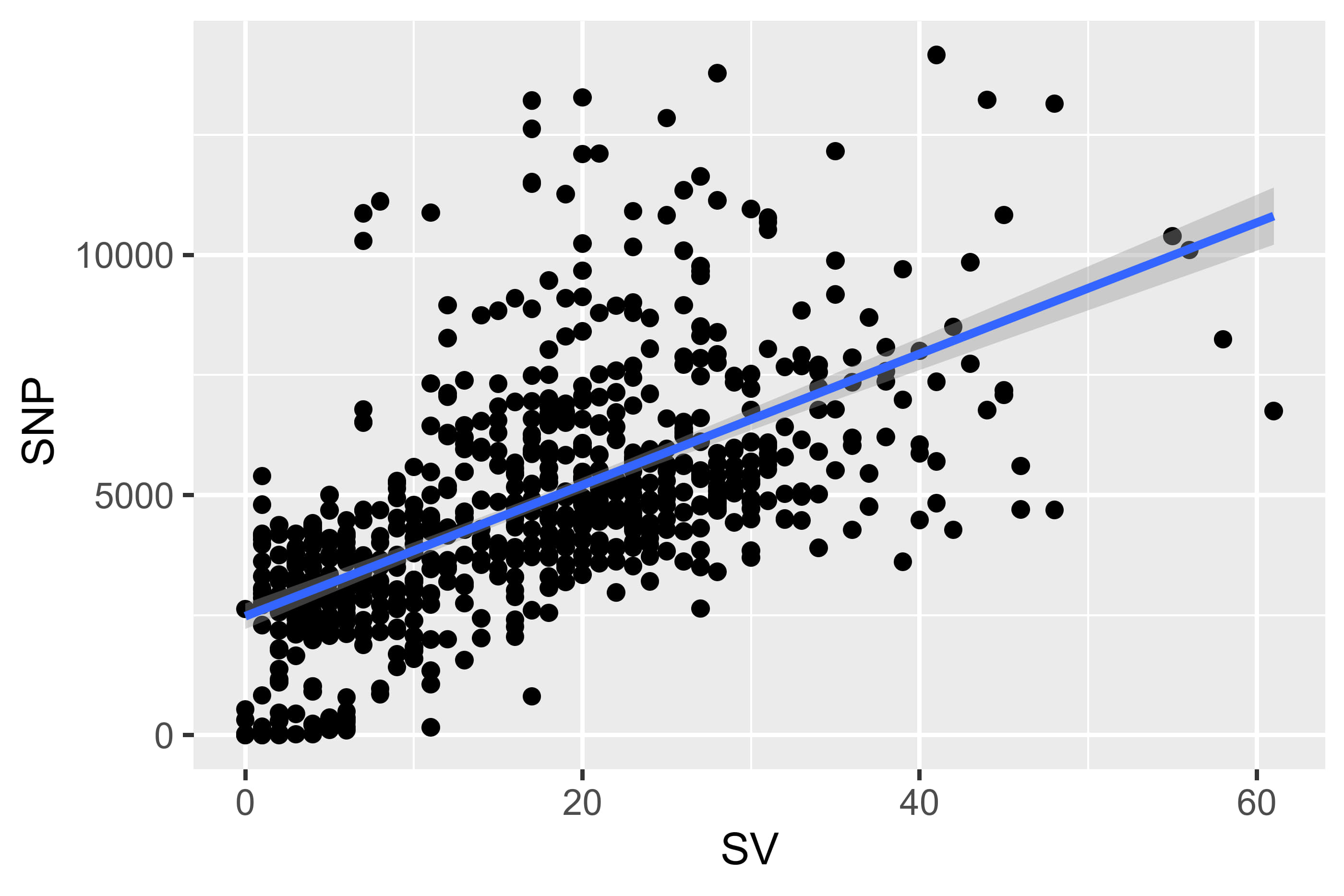


**Fig. S3** Correlation between the density of SNPs and SVs.


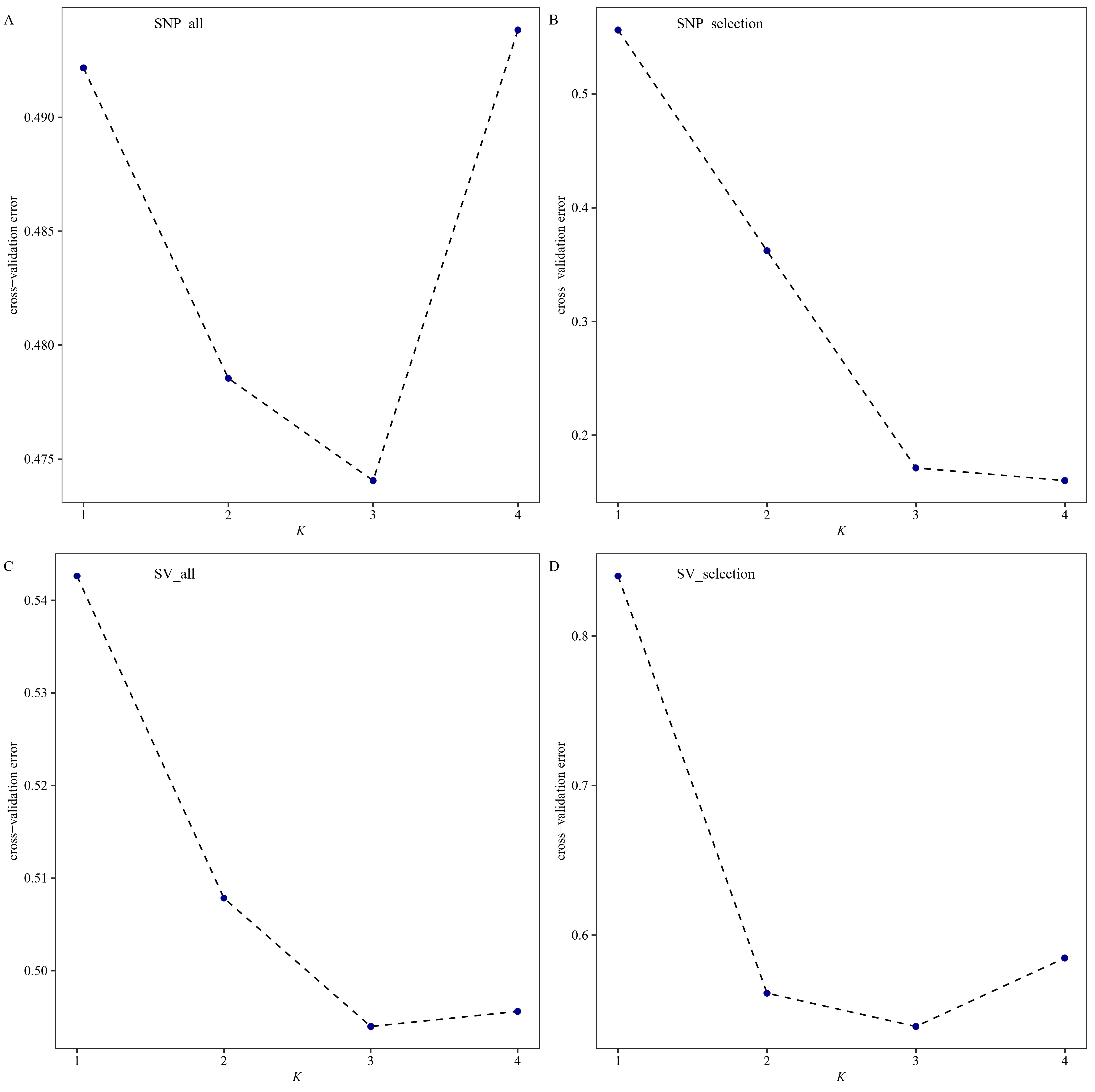


**Fig. S4** Cross validation error. **A** Cross validation errors for all SNPs. **B** Cross validation errors for selected SNPs. **C** Cross validation errors for all SVs. **D** Cross validation errors for selected SVs.


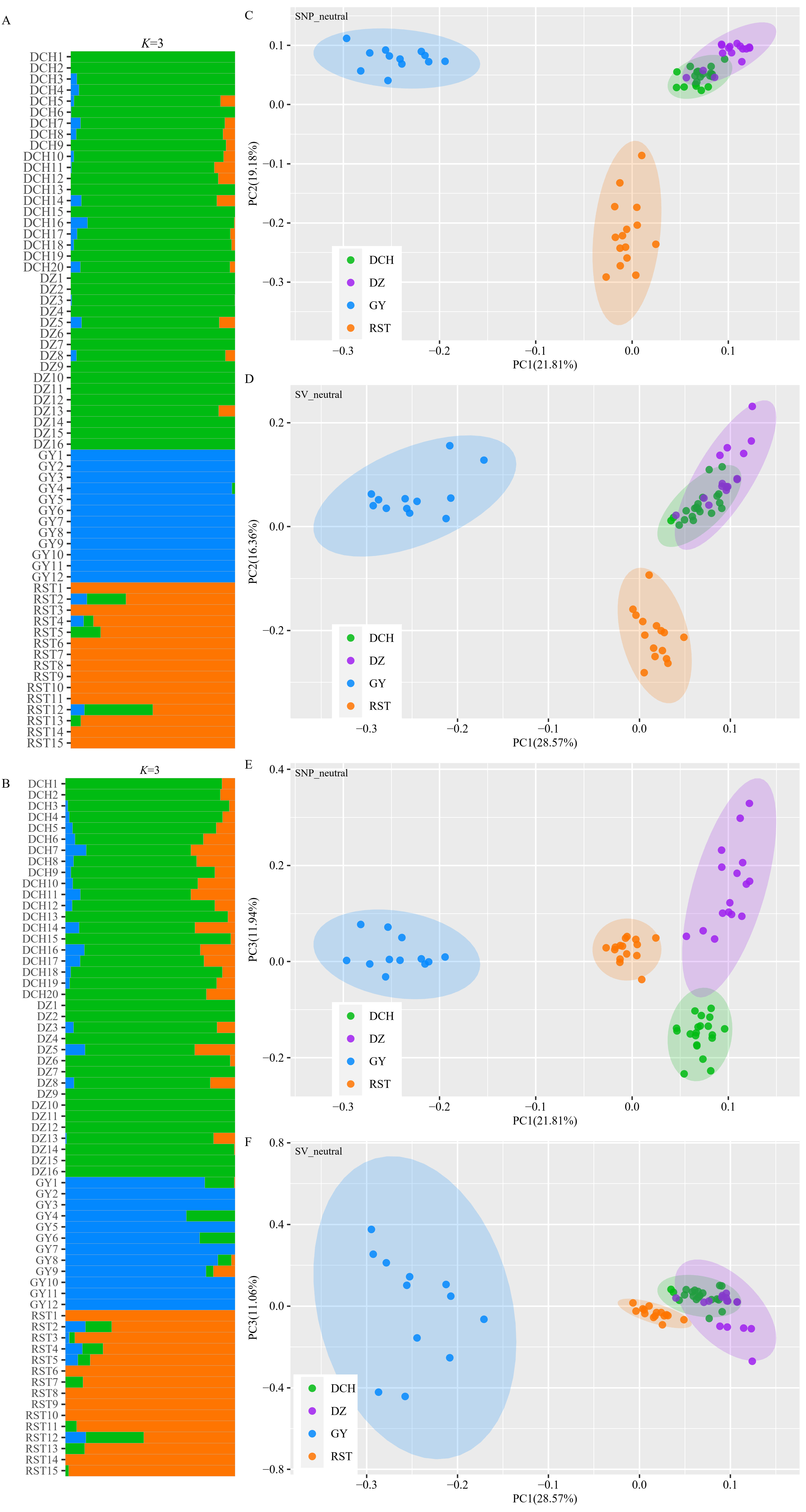


**Fig. S5** Population structure analyses of *Rhododendron griersonianum* using neutral loci. Admixture plots of neutral SNPs at *K* = 3; (B) Admixture plots of neutral SVs at *K* = 3; (C, E) Principal component analysis (PCA) plots based on neutral SNPs; (D, F) PCA result based on neutral SVs. DCH = Dachahe, DZ = Danzha, RST = Reshuitang, GY = Guyong.
